# Functional and Numerical Responses of the Predatory Mite, *Neoseiulus longispinosus*, to the Red Spider Mite, *Oligonychus* Coffeae, Infesting Tea

**DOI:** 10.1673/031.012.12501

**Published:** 2012-11-01

**Authors:** Vattakandy jasin Rahman, Azariah Babu, Amsalingam Roobakkumar, Kandasamy Perumalsamy

**Affiliations:** Entomology Division, UPASI Tea Research Foundation Tea Research Institute, Nirar Dam (BPO), Valparai 642 127 Coimbatore (Dt), Tamil Nadu, India; ^#^ Current address: Department of Entomology, Tocklai Experimental Station, Tea Research Association, Jorhat-785 008, Assam, India

**Keywords:** biocontrol, handling time, oviposition, pest, predation

## Abstract

Functional and numerical responses of the predatory mite, *Neoseiulus longispinosus* (Evans) (Acari: Phytoseiidae) to the red spider mite, *Oligonychus coffeae* Nietner (Acari: Tetranychidae), infesting tea were determined in a laboratory on leaf discs. Prey consumption increased with increases in temperature and prey density. Handling time decreased and successful attack rate increased with increased temperature. *N. longispinosus* was more voracious on larvae and nymphs than on adults of *O. coffeae*. Handling time was higher on adult females than on larvae. Rate of predation leveled off at temperatures greater than 25° C. Functional responses to prey density at six temperatures and to each life stage of *O. coffeae* approximated the Holling type II model. The oviposition rate increased with prey consumption and temperature. On average, a predator consumed 1.62 adult female prey for every egg it laid. With a fixed number of prey available, predation rate per predator decreased with increased predator density.

## Introduction

Functional and numerical responses give data on the biocontrol efficiency of natural enemies on a particular pest. The functional response of predators describes the relationship between the numbers of prey attacked at different prey densities. Numerical response is defined as the change in a predator's reproductive output at varying prey densities. Since the functional response is an important aspect in the dynamics of a predator-prey relationship and a major component of a population model, it has been used to predict the mechanisms underlying predator-prey behavior to improve the practical predictive potential of predator candidates for biocontrol (Sepúlveda and Carrillo 2008). Holling (1959a; 1959b; 1961) described three types of functional response curves: (1) a linear rise in prey consumption and the rate of reproduction to a plateau (type I), (2) a cyrtoid curve rising at a decreasing rate to a plateau (type II), and (3) a sigmoid curve with a positive accelerating rate up to an inflection point and thereafter a diminishing rate up to the plateau (type III). The basic factors that affect the functional response are: time of exposure of predator to prey, search rate, identification, capture, and prey consumption (Holling 1959b). The ability of the predator to control prey population is influenced by several factors, such as developmental response, utilization of alternative prey, behavioral patterns in response to the predator's own density, and intra-guild interaction (Lester and Harmsen 2002). The present study reports the functional and numerical responses of *Neoseiulus longispinosus* Evans (Acari: Phytoseiidae) on *Oligonychus coffeae* Nietner (Acari: Tetranychidae) in a laboratory. A laboratory experiment was also conducted to study the effect of mutual interference of predators on searching efficiency in the same feeding area.

While considering the pest scenario of tea in south India, the red spider mite, *O. coffeae* deserves special attention because of its widespread occurrence in all the tea growing areas causing about 18% crop loss (Muraleedharan et al. 2005). High incidence of this mite may occur in dry weather. *O. coffeae* infests the upper-surface of leaves, and, as a result of feeding on chlorophyll, the infested plants defoliate, causing the death of bushes and losses in crops. There is an increasing trend of switching over to integrated pest management practices in tea plantations because of the negative effects of synthetic pesticides used for the control of tea pests. Continuous use of synthetic pesticides causes a disruption of the natural balance between crop, pests and predators, the proliferation of pesticide resistant generations of pests, and undesirable residues in the tea (Rahman et al. 2012). Recent surveys conducted in the south Indian tea plantations (Vandiperiyar and Munnar (Idukki, Kerala), Coonoor and Kotagiri (The Nilgiris, Tamil Nadu), and Anamallais (Coimbatore, Tamil Nadu)) revealed the presence of a predatory mite, *N. longispinosus*, feeding on all stages of *O. coffeae*. Being major predators of Tetranychids and Eriophyids, Phytoseiid mites receive attention in agricultural pest control. The predatory mite *Scapulaseius newsami* (Evans) was introduced from Malaysia to India in 1961 for the control of *Calcarus carinatus, Brevipalpus californicus*, and *Oligonychus coffeae* in tea (Sankaran 1974). Zhang et al. (1999) reported the control potential of *N. longispinosus* on *Schizotetranychus nanjingensis*, a pest of bamboo. *N. longispinosus* was evaluated as a bio control agent (Zhang et al. 1998) and a mass release was done in bamboo forests (Zhang et al. 2000). It was reported as a promising biocontrol agent against citrus yellow mite, *Eotetranychus cendanai* (Thongtab et al. 2001), and two spotted spider mite, *Tetranychus urticae*, on strawberry (Kongchuensin et al. 2001).

Since there is little information on the predator-prey relationship between *N. longispinosus* and *O. coffeae*, an attempt was made to document the functional and numerical responses as well as the mutual interference response of *N. longispinosus* with *O. coffeae* as the prey. Data on functional responses were fitted to Holling's disc equation (Holling 1959a). Data on the numerical responses and oviposition at different temperatures were fitted to regression equations. The data on the response of predator to predator density were fitted to Hassell and Varley's (1969) equation. The values of each experiment can be retrieved from these equations.

## Materials and Methods

### Stock colonies of *O. coffeae* and *N. longispinosus*

Both *O. coffeae* and *N. longispinosus* were collected from the United Planters’ Association of Southern India tea experimental farm (Valparai, Coimbatore, Tamil Nadu, India) and used to start the stock colony. Adult red spider mites were transferred onto healthy tea leaves placed on a moist cotton pad, which was laid on the top of a sponge (0.5 inch thick) in plastic trays (42 × 30 × 6.5 cm). Water was added to the rearing unit when necessary to keep the cotton moist and thereby to prevent the leaves from drying.

Deteriorating leaves with red spider mite were cut and placed dorsal surface down on new leaves arranged as described previously to maintain the colony. Gravid females of *N. longispinosus* were collected from the red spider mite infested fields and reared on infested tea leaves placed on a moist cotton pad, which was kept in Petri plates (9 cm diameter). Cotton was kept oversaturated with water to prevent the possible escape of predatory mite. Petri plates were examined every two days to check over-population of the predator, and the leaves without enough spider mites to sustain the predatory mites were cut into small pieces and placed on new red spider mite infested tea leaves in Petri plates. Both the spider mite and predatory mite colony was maintained at 25±2° C and 75 ± 10% RH.

### General experimental methodology

The experimental arenas consisted of freshly excised tea leaf discs (2 cm in diameter) placed on moist cotton in Petri plates. Experiments on the functional response to different prey stages, the numerical response to different prey densities, and the response of predators to predator density were conducted at 25° C and 75 ± 5% RH. The photoperiod for all experiments was 14:10 L:D. Consumed prey in the arena of experiments on functional response were removed at each observation without destructing the web formed by spider mite, and the constant number of prey was maintained by adding new ones till the termination of the experiment. All experiments were conducted for seven consecutive days, and the mean values of all replications on each day were summed and averaged to get the final results of the experiment. The data from the first day of each experiment were excluded from the final analysis to dismiss the possibility of unusual response of the sample of predatory mites to an abrupt change in temperature, life stage of the prey, and prey density from that of stock colony.

### Functional response at different temperatures

Adult females of *N. longispinosus* were transferred from the stock colony onto an excised tea leaf with prey mites and allowed to lay eggs for 12 hours. Then, adults were removed, and the eggs of *N. longispinosus* were allowed to complete their post-embryonic development on the leaf. After mating, females were separated and used in the bioassays when they were 6 days old after hatching. They were starved for 24 hours on tea leaves arranged as described for the rearing of predatory mite. Each leaf disc was then inoculated with 1, 5, 10, 15, 20, 25, or 30 adult females of *O. coffeae*, and a single gravid female of *N. longispinosus* was introduced on each leaf disc. Experiments were conducted at 10, 15, 20, 25, 30, and 35° C with five replications for each experiment. The number of prey consumed per predatory mite was recorded every 24 hours for seven consecutive days.

### Functional response to different prey stages

The experimental methods and density of prey were similar to the above except that the functional responses of different life stages of *O. coffeae* (egg, larva, protonymph, deutonymph, and adult female) at 25° C were observed. Experiments on each life stage were replicated five times. The number of prey consumed per predatory mite was recorded every 24 hours for seven consecutive days.

### Numerical response to different prey densities

The numerical response of *N. longispinosus* to various densities of *O. coffeae* was studied in terms of the rate of oviposition of an individual predator to the rate of predation. A six-day-old gravid female predatory mite was starved for 24 hours and confined on a leaf disc with adult female *O. coffeae* at a density of 1, 3, 6, 9, or 12. The number of prey consumed and number of eggs laid by the predator were counted everyday for seven consecutive days with six replicates per prey density. Consumed prey were replaced daily to maintain the original number, and the eggs laid by the predator were removed every day.

### Numerical response at different temperatures

Ten adult red spider mites were confined in the experimental arena. A six-day-old gravid female predator was introduced onto the leaf. Experiments were conducted at 10, 15, 20, 25, 30, and 35° C. The number of eggs laid by the predator was recorded everyday for seven consecutive days with six replicates for each temperature. Dead prey were replaced with new ones every day.

### Response of predator to predator density

The influence of the mutual interference of predators on prey consumption was evaluated in the laboratory. The number of prey was fixed as 40 females of *O. coffeae* per leaf disc. Adult female predatory mites were introduced to different leaf discs at the density of 1, 3, 5, 7, or 9. The number of prey consumed was counted 24 hours after the start of the experiment. There were ten replicates for each predator density.

### Data analysis

Data were analyzed in Microsoft Excel (http://www.microsoft.com/). The functional responses were determined by fitting the data to the Holling disc equation (Holling 1959a), N_a_/P = aNT/1 + aT_h_N, where *N_a_* is the number of successful attacks per predator (*P*) during a specific time period (*T*), which in this is case 1 day, *N* is the initial density of the prey, and *a* and *Th* are the rate of successful attacks and the time required to handle the prey respectively. Handling time is defined as the time that the predator requires to pursue, kill, and digest the prey (Holling 1963). The parameters *a* and *Th* were calculated using a linear regression technique where 1/*Na* was regressed on 1/*N. a* is the reciprocal of the slope and *Th* is the intercept. The *a/Th* value indicates the effectiveness of predation. Maximum predation rate (*K*) was calculated as *T/Th*.

**Table 1.**
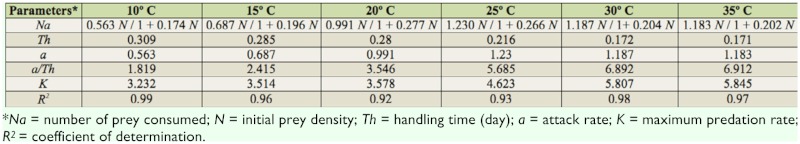
Functional response *of Neoseiulus longispinosus* adult females to *Oligonychus coffeae* adults at different temperatures.

The results of numerical responses and oviposition at different temperatures were fitted to regression equations. Different regression curves tested to fit the data are presented in this paper. The regression model whose R^2^ value was closer to 1 was selected to fit the data.

Since the relationship between searching efficiency and predator density is linear, the data of mutual interference of predators were fitted to Hassell and Varley's (1969) empirical model, log *E* = log *Q - m* log *P*; where *E* is the measure of searching efficiency, *P* is the predator density, *Q* is the quest constant (the value of *E* when *P* = 1), and *m* is the interference constant (the slope of the regression of log *E* on log *P*). Searching efficiency (E, a measure of the per capita predation when there is mutual interference between predators in the same feeding area) was calculated as 1/*P* log_e_ [*N/*(*N* - *N_a_*)], where *N* is the initial prey density, and *N_a_* is the number of prey consumed. Though a regression analysis of log *E* on log *P* to obtain *m* is not valid since *P* is used in the calculation of *E, m* could be significant if the corresponding value of *r* (regression coefficient) is significant (Varley et al. 1973).

## Results

### Functional response at different temperatures

The functional responses of predators at six different temperatures approximated the Holling type II model (Table 1) by a cyrtoid curve rising at a decreasing rate to a plateau. As the temperature increased, handling time (*Th*) decreased and successful attack rate (a) increased while a leveled off at temperatures greater than 25° C. The higher values of *a/Th* recorded at the temperatures above 25° C indicate that *N. longispinosus* was more effective against *O. coffeae* at those temperatures.

### Functional responses to different prey stages

A cyrtoid curve was observed in the case of functional responses of predators to different prey stages of *O. coffeae*; therefore, it was fitted to the Holling type II model (Table 2). *a/Th* values indicate that *N. longispinosus* was most effective against larvae and nymphs of *O. coffeae*.

### Numerical responses to different prey densities

**Table 2.**
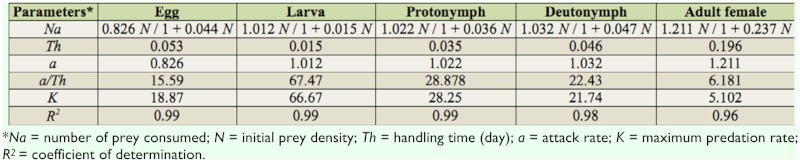
Functional response of *Neoseiulus longispinosus* adult females to different stages of *Oligonychus coffeae*.

**Figure 1.**
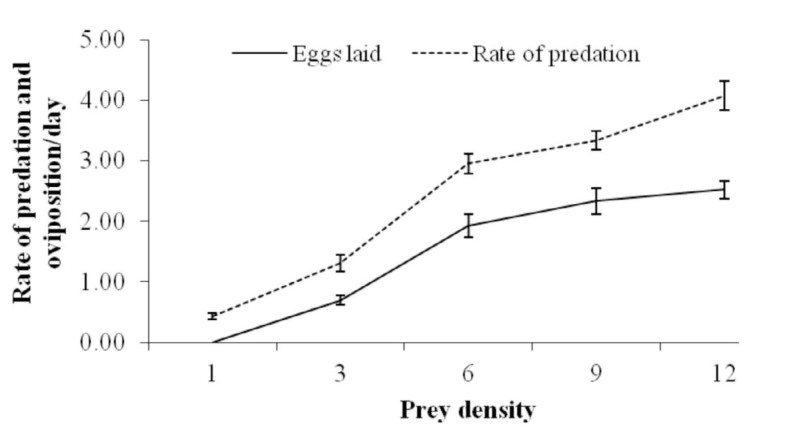
Numerical responses (mean ± SE) of *Neoseiulus longispinosus* female to different density of *Oligonychus coffeae* females. High quality figures are available online.

**Figure 2.**
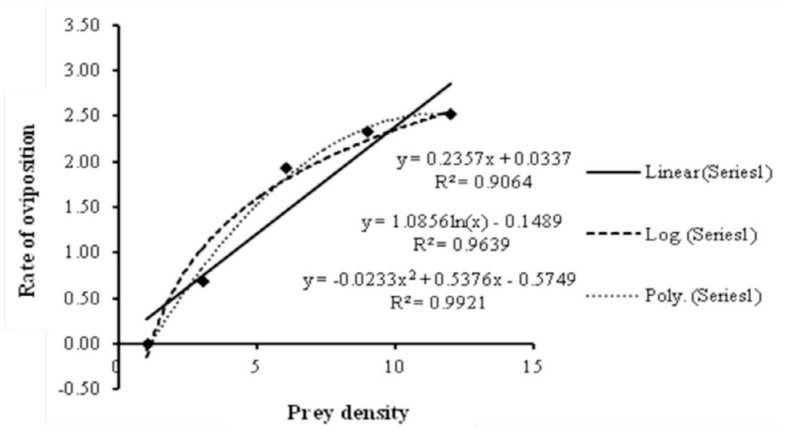
Regression models for the relationship between prey density and rate of oviposition of *Neoseiulus longispinosus*. High quality figures are available online.

The number of eggs laid per predator per day (y) was found to increase, with a gradual leveling off, with prey density (x) (Figure 1). The relationship can be expressed by the equation y = -0.023x^2^ + 0.537x - 0.574, (*R^2^* = 0.992) (Figure 2). At the density of 12 *O. coffeae* females per leaf disc, *N. longispinosus* laid a maximum of 2.52 eggs per day. No eggs were laid at the density of 1 red spider mite per leaf.

**Figure 3.**
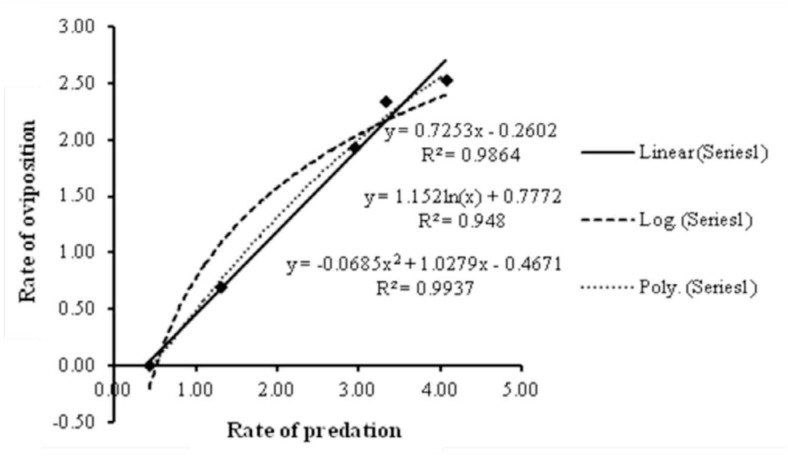
Regression models for the relationship between rates of predation and oviposition of *Neoseiulus longispinosus*. High quality figures are available online.

Oviposition rate (y) increased linearly with predation rate (x) (Figure 1); this relationship can be expressed by the equation, y = 0.725x - 0.26, (*R^2^*= 0.986) (Figure 3).

### Numerical response at different temperatures

**Figure 4.**
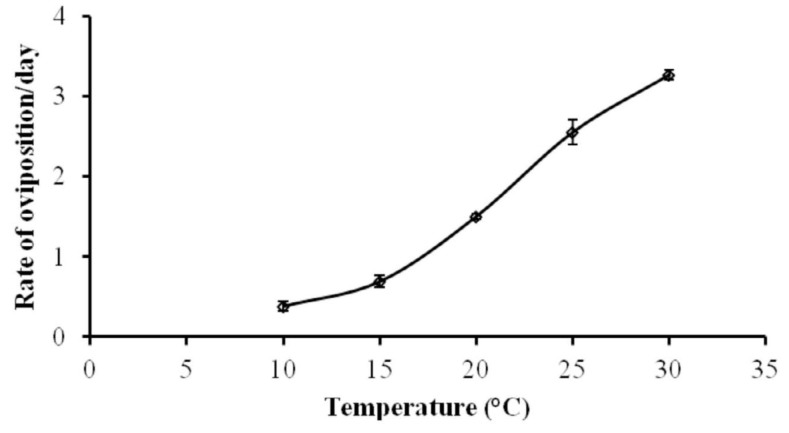
Numerical responses (mean ± SE) of *Neoseiulus longispinosus* adult females to *Oligonychus coffeae* adults at different temperatures. High quality figures are available online.

**Figure 5.**
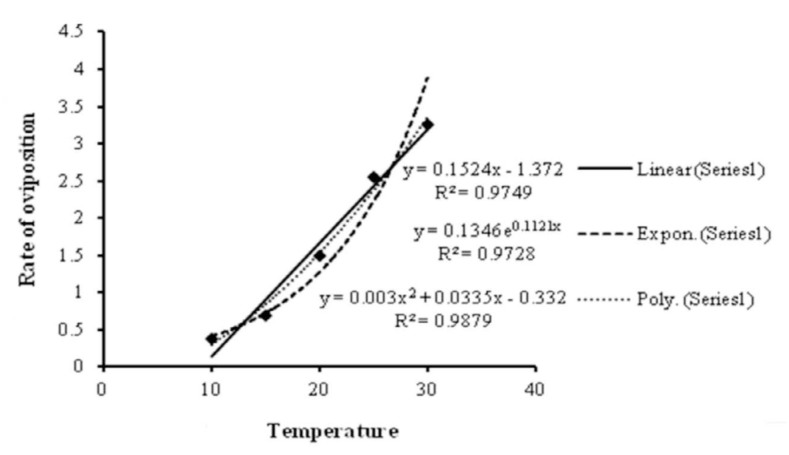
Regression models for the relationship between temperature and rate of oviposition of *Neoseiulus longispinosus*. High quality figures are available online.

The rate of oviposition increased with an increase in temperature (Figure 4). This relationship can be expressed by the equation y = 0.003X^2^ + 0.033X - 0.332, (*R*^2^ = 0.987), where *y* is the number of eggs laid by a predator per day and *x* is the temperature (Figure 5).

### Response of predators to predator density

Predation rate per predator decreased with an increase in predator density with a constant number of prey (40) available. The per capita rate of predation (per day) of *N. longispinosus* females on *O. coffeae* females decreased from 5.5 to 2.01in relation to the predator density increasing from 1 to 9. The relationship between searching efficiency and predator density was found to be linear, which can be expressed by the equation log *E* = log 0.148 – 1.481 log *P* (*R^2^*= 0.993).

## Discussion

In this study, the predatory mite *N. longispinosus* was found to consume all stages of *O. coffeae*. The maximum predation rate is limited by an upper asymptote defined by the ratio *T/Th* (*K*) (Hassell 1978), which was higher at 30 and 35° C for *N. longispinosus*. This result is desirable for the biocontrol of *O. coffeae* because the maximum daily temperature in the summer, during which *O. coffeae* is present, fluctuates around 30° C in south Indian tea plantations. When tested with different prey stages, larvae and nymphs of *O. coffeae* were more consumed than other stages; therefore, *K* value was higher for larvae and nymphs. Blackwood et al. (2001) observed a strong preference of *N. longispinosus* for the egg stage of *T. urticae*. However, Ibrahim and Palacio (1994) noted that *N. longispinosus* preferred larvae and nymphs of *Tetranychus urticae* to eggs. The predatory mites *N. californicus* (Canlas et al. 2006) and *Kampimodromus aberrans* (Kasap and Atlihan 2010) also showed preference for larvae when fed *T. urticae*.

The preference for younger stages will be helpful in preventing the proliferation of pest population. Predation rate was found to increase with increased density of prey. The reason for increased predation rate at higher prey densities may be the interference or disturbance by the prey as noticed in *Amblyseius largoensis* (Muma), *Euseius corcordis* (Chant), and *Galendromus* (= *Typhlodromus*) *helveolus* (Chant) (Sandness and Mc Murtry 1970). At higher prey densities, predators may spend less time on individual prey because prey accidentally bumping into a feeding predator may cause the predator to abandon the prey it is eating and attack another. The bumping of prey into each other may cause prey to move, thereby increasing the chance of an encounter with a predator. This higher prey density results in wasteful killing (Metz et al. 1988), as a predator may partially eat multiple prey instead of eating one whole prey. Predation rate reaches a plateau at higher prey densities due to factors such as satiation (Sabelis 1985). The number of prey consumed by *N. longispinosus* increased with increased density of *T. urticae* and leveled off at a prey density of 40 per predator (Ibrahim and Rahman 1997). The density dependant interference or defensive behavior of spider mites may also have caused the reduced rate of successful attacks.

The oviposition rate of *N. longispinosus* appears to depend on temperature and the quality of prey taken as food. The rate of oviposition increased with increased prey consumption. Oviposition correlates with predation because phytoseiid mites allocate a major fraction of food ingested to egg production (Sabelis and Janssen 1994). At the density of one prey per leaf, predators survived but hardly laid any eggs. On average, one egg was laid for every 1.62 prey eaten. The increase in oviposition in response to the increase in prey density adds to the effectiveness of biological control by causing an increase in predator population. The rate of oviposition increased with an increase in temperature from 10 to 30° C. In a previous study, it was shown that *N. longispinosus* could not survive at temperatures above 35° C (unpublished data).

An increase in the population size of a predator may cause a decrease in prey consumption. Mutual interference denotes the adverse influence of predator density on the instantaneous success of individual predator (Arditi and Akcakaya 1990). However, spatial complexity and aggregation also have roles in predator-prey interactions (Hassell and May 1973; Free et al. 1977). Under field conditions, interference probably leads to the emigration of individual predators from regions of high parasite density (Rogers and Hassell 1974). Begon et al. (1996) studied mutual interference with an unlimited number of prey. But 40 prey is large for predatory mites as far as their predation rate is concerned. So the effects of mutual interference will not be mixed up with the effects of competition for food. In the present study, predator interference affected the rate of prey consumption. However, the natural dispersal of predators to explore for more prey will reduce this interference behavior among predators in field.

Results reported here form a database for further studies on the feasibility to incorporate *N. longispinosus* in integrated pest management programs against *O. coffeae* in tea.
